# Structural Biology and Structure–Function Relationships of Membrane Proteins

**DOI:** 10.3390/biology10030245

**Published:** 2021-03-22

**Authors:** Isabel Moraes, Andrew Quigley

**Affiliations:** 1 National Physical Laboratory, Hampton Road, Teddington TW11 0LW, UK; 2 Membrane Protein Laboratory, Diamond Light Source Ltd., Harwell Science and Innovation Campus, Didcot OX11 0DE, UK; 3 Research Complex at Harwell (RCaH), Harwell Science and Innovation Campus, Didcot OX11 0FA, UK

To understand the biological complexity of life, one needs to investigate how biomolecules behave and interact with each other at a molecular level. Molecular structures, such as proteins, are known to be vital mediators in cellular processes. Like state-of-the-art machines, these proteins perform specific tasks with a very high precision. 

Specifically located across biological membranes, integral membrane proteins perform a large array of functions, all of which are critical to a cell’s existence and maintenance. It is predicted that more than one quarter of the human genome codes integral membrane proteins [1]. Like their soluble counterparts, membrane proteins’ three-dimensional fold/structure is intimately associated with their function. Mutations and misfolding of membrane proteins are related to many disorders, including cancer, diabetes, obesity, and neurodegenerative diseases such as Alzheimer’s and Parkinson’s. Consequently, integral membrane proteins are considered important therapeutic targets. Indeed, around 60% of the drugs currently on the market target membrane proteins, with G-protein coupled receptors, ion channels, and transporters representing the largest groups [2,3]. Furthermore, membrane proteins are also important in understanding human–parasite interactions, including bacteria and viruses. A strong example is the present outbreak of COVID19 caused by the severe acute respiratory syndrome coronavirus-2 (SARS-CoV-2). The rapid deposition of atomic resolution structures of the viral integral membrane protein, known as the spike protein, with another integral membrane protein, known as the human angiotensin-converting enzyme-2 receptor, which is highly expressed in lung and heart tissues, has significantly contributed to the understanding of the molecular mode of action of SARS-CoV-2. This has enabled the development of many pharmaceutical agents that could provide treatments for COVID19 [4,5,6]. Today, structural information on membrane proteins with an atomic resolution, combined with functional assays and computational approaches such as molecular dynamics simulations, are critical to biology and modern pharmaceutical drug discovery. 

Structural biology has played a major role in the study and understanding of relationships between the protein structure, function, and dynamics at an atomic level. In the last few decades, biophysical techniques such as x-ray crystallography, single particle electron microscopy (EM), nuclear resonance microscopy (NMR), and neutron diffraction, combined with developments in proteomics and genomics, have been at the forefront of protein structure determination. However, the path to membrane protein structure determination has been long and arduous. More than 35 years has passed since the first membrane protein structure was solved [7]. Many Noble prizes regarding advances in the field have been awarded. Nevertheless, only 3% of all protein structures in the wwPDB (Worldwide Protein Data Bank) represent unique membrane proteins (as reported in https://blanco.biomol.uci.edu/mpstruc/ on 18 March 2021) and many key questions are still unanswered. This means that a great deal of work still needs to be conducted. Understanding how membrane proteins interact with biological membranes (i.e., the role of lipids in protein activity) and other proteins will uncover the molecular mechanisms behind signal transduction, drug/solute transport, channel gating and cell adhesion. 

In this *Special Issue*, a series of articles written by international leaders in the field highlight the importance of membrane protein structural studies and how modern structural biology approaches are contributing to their pace and development. 

Amy E. Danson, Alex McStea, Lin Wang, Alice Y. Pollitt, Marisa L. Martin-Fernandez, Isabel Moraes, Martin A. Walsh, Sheila MacIntyre and Kimberly A. Watson [8] address and explore the effect of several mutations on the *Chlamydia* major outer membrane protein (MOMP). *Chlamydia pneumoniae* is a Gram-negative bacterium that is responsible for several human respiratory diseases, including specific chronic inflammatory diseases. Contrary to other Gram-negative bacteria, in *Chlamydia*, the peptidoglycan layer is absent for much of its lifecycle. Instead, it is a group of cysteine-rich outer membrane proteins that provide a sort of strong mesh-like structure, known as the chlamydial outer membrane complex (COMC), which protects against bacterium cell damage. The *Chlamydia* MOMP is a porin β-barrel membrane protein that compromises around 60% of the COMC. Containing nine cysteine residues, studies have suggested that MOMP has a major role in forming a number of intermolecular disulphide bonds with other MOMPs in the outer membrane, and is thus an important primary target for vaccine development. Nevertheless, its structural characterisation has not been conducted and most of its mechanisms of action are still unknown. In this work, the authors created seven MOMP cysteine/alanine mutants with the aim of studying the possible disulphide bonds between the MOMP and other proteins in the COMC network. Super-resolution fluorescence microscopy methods such as epifluorescence, total internal reflection fluorescence microscopy (TIRF) and direct stochastic optical reconstruction microscopy (dSTORM) were used to identify and assess the localisation of the recombinant MOMP in the whole *E. coli* cells, as well as to measure the clustering behaviour of the different mutants. The authors conclude by proposing a cysteine-rich pocket hypothesis; that is, the COMC formation and stability might be due to a general MOMP cysteine-rich region, rather than specific single cysteine residues.

Vivien Yeh, Alice Good, and Boyan Bonev [9] beautifully review the current capabilities of both solution and solid-state nuclear resonance microscopy (NMR) techniques applied to membrane protein characterisation and structural studies. Solution and solid-state NMR are powerful microscopy approaches that have been part of the structural biology toolbox for a long time. However, recent developments in both techniques have opened a new range of opportunities that go from higher sensitivity signal measurements to studies of lipid environments. While conventional solution NMR provides important structural information on membrane proteins that have been solubilised in surfactants such as detergents, solid-state NMR goes a step further and provides structural information on samples where membrane proteins are embedded in lipid bilayers. As a result, solid-state NMR presents the advantage of being able to not only measure conformational changes of membrane proteins in the presence of different lipid compositions, but also to provide information on the protein orientation within the membrane. In this review, the authors provide a good selection of successful examples in which NMR was employed to characterise the structure–function relationships of membrane proteins. In addition, there is also a very good description of sample preparation, including the production of membrane mimetic systems. Finally, like any other biophysical method, each NMR approach has its own application and limitations; these are also discussed in detail. 

Rosana I. Reis and Isabel Moraes [10] explore and demonstrate a new approach for investigating and validating the assembly of membrane proteins into nanodiscs for functional and structural studies. Once removed from their biological membrane, membrane protein aggregation and stability have been two of the major challenges associated with in vitro studies. The loss of native lipids during the solubilisation and purification processes often compromises the structural integrity of the membrane protein in the study. Therefore, in recent years, the use of lipidic nanodisc technology for membrane protein studies has become exceptionally popular, as it provides a superior native-like membrane environment than the use of detergent micelles or liposomes. In addition, this technology has proven to be very successful in many applications that range from functional studies to biophysical applications, such as cryo-EM, solid-state NMR and surface plasmon resonance (SPR). However, the nature and ratio between lipid mixtures; the ratio between the membrane scaffold protein (MSP) and lipids; and the ratio between lipids, MSP and the target proteins need to be determined empirically, making the whole process laborious and time consuming. In this article, the authors propose the use of in situ dynamic light scattering as a high-throughput screening tool for assessing the best conditions for nanodisc assembly and protein incorporation. As proof of concept, the human G protein-coupled receptor A_2A_ is used.

James Birch, Harish Cheruvara, Nadisha Gamage, Peter J. Harrison, Ryan Lithgo and Andrew Quigley [11] provide a compressive review on the most recent advances in the field of membrane protein structural biology. The review takes the reader through each step in the process of solving the atomic resolution structure of a membrane protein. Particular emphasis is placed on the requirements for high quality membrane protein samples for cryo-electron microscopy and crystallography. Methods for determining the quality are described, along with examples from the authors’ own experience. A comparison of the requirements for both membrane protein crystallisation and cryo-EM is conducted. Finally, a perspective is given on the future of membrane protein structural biology and the drive towards understanding atomic resolution structures within the cellular context and the possibilities of an integrative structural biology-based approach.

José Edwin Neciosup Quesñay, Naomi L. Pollock, Raghavendra Sashi Krishna Nagampalli, Sarah C. Lee, Vijayakumar Balakrishnan, Sandra Martha Gomes Dias, Isabel Moraes, Tim R. Dafforn and Andre Luis Berteli Ambrosio [12] provide a detailed historical review focused on the human mitochondrial pyruvate transporters (MPC1 and MPC2). Here, they tell the story of the efforts to first identify and subsequently characterise the structure and function of these integral membrane proteins. The authors highlight a series of studies that have looked at the multimerisation of MPC1 and MPC2 and the effect of this on the human and yeast MPC function. Detergent-based studies point towards the importance of homo- and heterodimerisation and the requirement for cardiolipin, while MPC2 solubilised using styrene maleic acid co-polymers (SMALPs) may suggest that high-order complexes exist in more native-like environments. Although structures exist for the related bacterial semi-SWEET transporter, MPCs have not been investigated at an atomic resolution. High-resolution structures of MPCs will be important for understanding MPC domain organisation and the mechanism of pyruvate transport. The authors present initial 2D classifications from a cryo-EM study of MPCs solubilised in SMALPs, showing that we are moving towards an atomic resolution structure of humans’ MPCs.

In their in-depth review of ATP-binding cassette transporters from *Mycobacterium tuberculosis*, Marcelo Cassio Barreto de Oliveira and Andrea Balan [13] explore the current structural and functional understanding of this group of proteins. The authors highlight the main substrate classifications, functional roles, and corresponding structural organisation of both importers and exporters in a clear and engaging manor. Of particular note are the roles that ABC exporters play in *M. tuberculosis* virulence and resistance to antibiotics. Finally, the authors address the distribution of ABC transporters across *Mycobacterium*, before highlighting two novel ABC-like proteins that are important for Mammalian cell entry.

We hope that readers find this small collection of articles interesting. They shed light on the importance of understanding membrane protein structure–function relationships. Membrane protein structural studies are still associated with many challenges. Nevertheless, the future looks bright due to the many constant technical developments in the field of structural biology—modern structural biology, as it is called today! These technical developments, along with integrative approaches to structure–function relationships of membrane proteins that span atomic to cellular length-scales on micro- to nano-second time-frames, will revolutionise our understanding of membrane protein disease biology. More and more membrane protein structures are being determined, revealing their mechanisms of action. Looking forward and never giving up is the message that we would like readers to take away from this work! 

We thank all the authors for their time and efforts devoted to producing such high quality articles.

## Data Availability

Not applicable.
